# Antimicrobial Peptide Evolution in the Asiatic Honey Bee *Apis cerana*

**DOI:** 10.1371/journal.pone.0004239

**Published:** 2009-01-21

**Authors:** Peng Xu, Min Shi, Xue-xin Chen

**Affiliations:** Institute of Insect Sciences, Zhejiang University, Hangzhou, Zhejiang, China; Massachusetts General Hospital, United States of America

## Abstract

The Asiatic honeybee, *Apis cerana* Fabricius, is an important honeybee species in Asian countries. It is still found in the wild, but is also one of the few bee species that can be domesticated. It has acquired some genetic advantages and significantly different biological characteristics compared with other *Apis* species. However, it has been less studied, and over the past two decades, has become a threatened species in China. We designed primers for the sequences of the four antimicrobial peptide cDNA gene families (*abaecin*, *defensin*, *apidaecin*, and *hymenoptaecin*) of the Western honeybee, *Apis mellifera* L. and identified all the antimicrobial peptide cDNA genes in the Asiatic honeybee for the first time. All the sequences were amplified by reverse transcriptase-polymerase chain reaction (RT-PCR). In all, 29 different *defensin* cDNA genes coding 7 different defensin peptides, 11 different *abaecin* cDNA genes coding 2 different abaecin peptides, 13 different *apidaecin* cDNA genes coding 4 apidaecin peptides and 34 different *hymenoptaecin* cDNA genes coding 13 different hymenoptaecin peptides were cloned and identified from the Asiatic honeybee adult workers. Detailed comparison of these four antimicrobial peptide gene families with those of the Western honeybee revealed that there are many similarities in the quantity and amino acid components of peptides in the abaecin, defensin and apidaecin families, while many more hymenoptaecin peptides are found in the Asiatic honeybee than those in the Western honeybee (13 versus 1). The results indicated that the Asiatic honeybee adult generated more variable antimicrobial peptides, especially hymenoptaecin peptides than the Western honeybee when stimulated by pathogens or injury. This suggests that, compared to the Western honeybee that has a longer history of domestication, selection on the Asiatic honeybee has favored the generation of more variable antimicrobial peptides as protection against pathogens.

## Introduction

The honeybee is a eusocial insect whose role in plant pollination is essential to global ecology. Through pollination of flowering plants, honeybees help maintain biodiversity and supply commodities such as honey, royal jelly, propolis, pollens, and wax. Publication of the genome sequence of the Western honeybee, *A. mellifera* L. has greatly facilitated genomic research on this economically important insect [1]. The discovery of a 100-million-year-old amber fossil pushes back the date that bees began pollinating flowers by millions of years [2], although species of *Apis* are much younger than this.. Honeybees defend their nest, brood and stored food through many complicated and efficient defensive behaviors [3], but populations have decreased rapidly in many countries in recent years. Explanations for this decline include global warming caused by the greenhouse effect, indiscriminate use of pesticides, reduction or extinction of plant species and other human-induced disturbances. In addition, infection by strong pathogenic microorganisms (such as viruses) or pests (such as mites) have been cited as important direct or indirect reasons for honeybee collapse [4]–[9]. Protection of the honeybee species is a worldwide concern.

The Asiatic honeybee, *A. cerana* Fabricius, plays an important role in maintaining the biodiversity of plants in China. *A. cerana* is still found in the wild, where it nests in tree holes, fallen logs, and crevices, but it is also one of the few bee species that can be domesticated. It has acquired some incomparable advantages in its long history of evolution compared with other introduced *Apis* species. It has adapted to adverse climatic conditions, and can survive extreme fluctuations in temperature and long periods of rainfall. For example, it can survive when the air temperature is as low as −0.1°C, a temperature lethal for the Western honeybee [10]. It is a natural host for, and can tolerate the mite, *Varroa destructor*, and the microsporidian, *Nosema ceranae*, both of which are the serious pests of the Western honeybee [11], [12]. Having coevolved with these parasites, the Asiatic honeybee exhibits more careful grooming behavior than the Western honeybee, and appears to have other more effective defenses against these parasites. The Asiatic honeybee can also effectively pollinate mountain plants and crops, especially the early flowering fruits and vegetables in high altitude regions, such as the Himalayan region of China where temperatures are too low for the exotic *A. mellifera* [10]. Nevertheless, over the last 20 years, it has become a threatened species in China for many reasons, most likely because of competition from the introduced Western honeybee, *A. mellifera*. More research is needed and urgent measures must be taken to protect the Asiatic honeybee. It is noteworthy that the Asiatic honeybee does not produce propolis, a highly valued resinous material that is used to seal small open spaces in the hive, and is believed to be involved in the honeybee defense system against pathogens in the hive.

Honeybees defend against many pathogens by producing antibiotic substances such as propolis and royal jelly [13], [14]. The innate immune system is the first line of defense against pathogens in plants and invertebrate animals, and it is also critical for vertebrate immunity before the acquired immune system generates a specific response [15]–[17]. Various antimicrobial peptides are the key elements of the insect immune system [18]–[20]. After the honeybees are infected by pathogens, four antimicrobial peptide families are synthesized, representing a broad spectrum of antimicrobial activity in the haemolymph. All of these are cationic peptides identified as: apidaecins [21], abaecin [22], hymenoptaecin [23] and defensin [24]. Recently, two structurally different defensin genes were cloned from *A. mellifera* [25]. Almost all of the honeybee antimicrobial peptides and the antimicrobial peptide genes are reported from the Western honeybee; however, little research has been conducted on the Asiatic honeybee. As an very ancient and important native honeybee species in China, many aspects of the species need to be explored. Here we examine the antimicrobial peptide gene families of the Asiatic honeybee and compare them with those of the Western honeybee.

## Results

### Analysis of *defensin* cDNA genes

**Figure 1:**
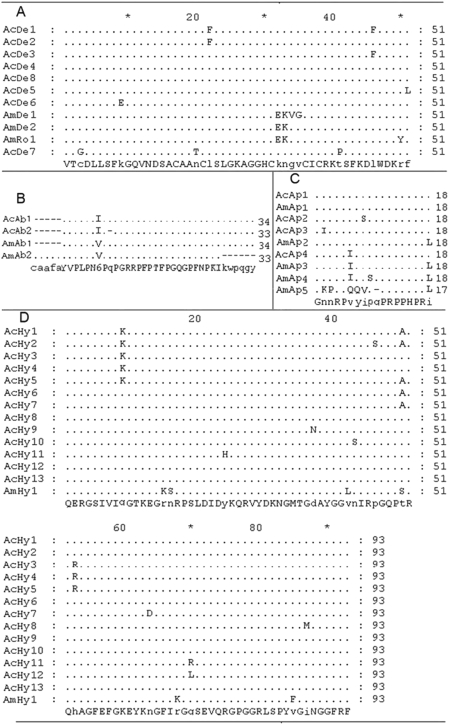
Alignment of mature peptides of the four antimicrobial peptide families of *A. cerana* and *A. mellifera*. (A): Alignment of mature peptides of honeybee defensins. AcDe1 to AcDe8 from the Asiatic honeybee; AmDe1, AmDe2 and AmRo1 from the Western honeybee, AcDe8, AmDe1, AmDe2 and AmRo1 downloaded from the NCBI website. (B): Alignment of mature peptides of honeybee abaecins. AcAb1 and AcAb2 from the Asiatic honeybee; AmAb1 and AmAb2 from the Western honeybee and downloaded from the NCBI website. (C): Alignment of mature peptides of honeybee apidaecins. AcAp1 to AcAp4 from the Asiatic honeybee; AmAp1∼AmAp5 from the Western honeybee and downloaded from the NCBI website. (D): Alignment of mature peptides of honeybee hymenoptaecins. AcHy1∼AcHy13 from the Asiatic honeybee; AmHy1 from the Western honeybee and downloaded from the NCBI website. Multiple sequence alignment performed using Clustal_X. Identical amino acids are in black dots. Different amino acids are shown in the alignment. Small dashes indicate gaps inserted for optimal alignment. Amino acid residues are numbered on the right.

In total, 29 different *defensin* cDNA sequences (*Defensin1∼Defensin29*, GenBank: EU727268∼EU727296) coding 7 different defensin peptides (termed as AcDe1∼AcDe7) were amplified and identified. All of these cDNA genes have high sequence identity with the *defensin* cDNA gene of the Western honeybee. Nearly two-thirds (20/29) of the cDNA genes coded the major peptide AcDe4, and one or two cDNA genes coded for defensin peptides AcDe1∼AcDe3 and AcDe5∼AcDe7. The major peptide AcDe4 was identical to peptide AcDe8 reported for the Asiatic honeybee *defensin* cDNA gene (GenBank: ABS10820, direct submission). All of the inferred mature peptide sequences are highly homologous with the Western honeybee two defensin peptides (AmDe1 coded by one *defensin* cDNA gene, GenBank: U15955, and AmDe2 coded by one *defensin* cDNA gene, GenBank: AAR01214) and the royalisin peptide (termed as AmRo1) [26] (Figure 1A). Compared with the Western honeybee defensin peptides, one to three amino acid substitutions exist in each defensin peptide of the Asiatic honeybee. Mutations occur between the two hydrophobic amino acids, Phe and Leu, in AcDe1, AcDe2, AcDe3 and AcDe5, which may not change the property and structure. One acidic amino acid (Glu) substitutes the basic amino acid (Lys) at position 9 in AcDe6, which decreases the isolectric point and may change antimicrobial properties. Three amino acid substitutions exist in AcDe7 as follows: one apolar amino acid (Gly) substitutes the polar amino acid (Cys) at position 3, one apolar amino acid (Pro) substitutes the polar amino acid (Thr) at position 41 and one natural mutation occurs at position 20 (Thr-Asn) in AcDe7 (Figure 1A). The first substitution may change the secondary structure of AcDe7, the second one would decrease the hydrophilic ability and may change other antimicrobial properties of AcDe7 and the last one may have no affects. There is one other unique Western honeybee defensin peptide (AmDe3, GenBank: NP 001011638), which is an inferred-peptide sequence for a recognized paralog of the definitive Western honeybee *defensin* gene [25]. It seems entirely unique compared with the amino acid sequences of other all defensins and royalisin. It has only 43 amino acids (VTCDVLSWQSKWLSINHSACAIRCLAQRRKGGSCRNGVCICRK), while all others have 51 amino acids, and more amino acid substitutions among them. Moreover, the ORF of the Asiatic honeybee *defensin* has one extra amino acid (Gly) at the C-terminus that may be amidated as in the mature antimicrobial peptide of *A. mellifera* defensin [24], [27].

**Figure 2:**
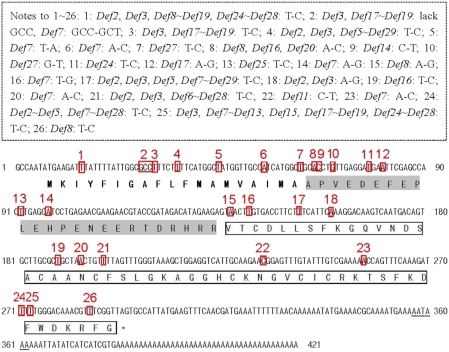
Nucleotide sequence and predicted amino acid sequence of *Defensin1* (GenBank Accession EU727268). The putative signal peptide is bolded, the proregion of the peptide is shadowed, and the mature peptide is indicated by an open box. The translational signal (TAG) is indicated by the star symbol. Restriction and poly (A) adenylation signals (AATAAA) are indicated by a wavy line. The variant loci are boxed and numbered in red. The nucleotide substitution sites in the precursor sequences between *Defensin1* and other *Defensin* genes are boxed with a dotted line. “*Defensin1∼Defensin 29*” is abbreviated to “*Def1∼Def29*”.

The Asiatic honeybee defensin precursor gene is composed of three parts: a pre-region or signal region (coding 18 or 19 amino acids), a pro-region (between signal and mature region, coding 24 amino acids) and a mature region (coding 52 amino acids) (Figure 2). In total 303 nonsynonymous substitutions (NS) and 451 synonymous substitutions (SS) exist in the 29 precursor genes, 55 NS and 116 SS in the pre-pro-region, and 136 NS and 97 SS in the mature region. Most of the NS occupy the mature region, and most of the SS are in the pre-pro-region. The *defensin* mRNA transcript continues for an additional 41–126 bases (excluding the PolyA) after the stop codon (Figure 2).

### Analysis of *abaecin* cDNA genes

In all, 11 different *abaecin* cDNA sequences (*Abaecin1∼Abaecin11*, GenBank: EU714043∼EU714053) coding 2 different abaecin peptides (termed as AcAb1 and AcAb2) were amplified and identified. Seven different cDNA genes coded for the major peptide AcAb1 and 4 different cDNA genes coded for peptide AcAb2. AcAb1 consists of 34 amino acids, while AcAb2 consists of 33 amino acids, and lacks the Gln at position 9 of Ab1 (Figure 1B). One natural mutation (Ile-Val) at position 7 exists in AcAb1 and AcAb2 compared with the Western honeybee abaecin peptide (termed as AmAb1 coded for by one Western honeybee *abaecin* cDNA gene, GenBank: NM_001011617) (Figure 1B), which may not change their structures and properties; one polar amino acid Gln is lacking in AcAb2, which would decrease the hydrophilicity of AcAb2. One polar amino acid Ser substitutes the apolar amino acid Ile at position 8 in AcAp2, and one apolar acid Ile substitutes the polar amino acid Asn. Both of these exist in the variable region, which would change the general antimicrobial spectrum while the conserved (constant) region remains the same. This would maintain general antimicrobial capacity [29], [33]. One other Western honeybee *abaecin* peptide (termed as AmAb2, direct submission GenBank: U15954) is a special peptide, consisting of 33 amino acids with 5 amino acids (CAAFA) extending at the N-terminal and 6 amino acids (KWPQGY). It is absent at the C-terminal compared with other honeybee abaecin peptides.

**Figure 3:**
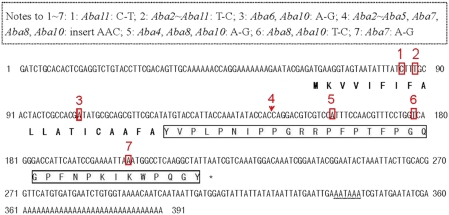
Nucleotide sequence and predicted amino acid sequence of *Abaecin1* (GenBank Accession EU714043). The putative signal peptide is bolded, and the mature peptide is indicated by an open box. The translational signal (TAA) is indicated by the star symbol. Restriction and poly (A) adenylation signals (AATAAA) are indicated by a wavy line. The variant loci are boxed or indicated in red by a short arrow, and numbered in red. The nucleotide substitution sites in the precursor sequences between *Abaecin1* and other *Abaecin* genes are boxed with a dotted line. “*Abaecin1∼Abaecin11*” is abbreviated to “*Aba1∼Aba11*”.

The Asiatic honeybee abaecin precursor gene is made up of two parts: a pre-region (coding 19 amino acids) and a mature region (coding 33 or 34 amino acids). It lacks a pro-region (Figure 3). In total, 22 NS and 48 SS exist in the 11 precursor genes, 3 NS and 6 SS in the pre-region and 4 NS and 10 SS in the mature region. The *abaecin* mRNA transcript continues for an additional 140 bases after the stop codon (excluding the PolyA) (Figure 3).

### Analysis of *apidaecin* cDNA genes

In all, 13 different *apidaecin* cDNA sequences (*Apidaecin1∼Apidaecin13*, GenBank: EU727255∼EU727267) coding 4 different apidaecin peptides (termed as AcAp1∼AcAp4) were amplified and identified. The apidaecin peptides from the Western honeybee are termed here as AmAp1∼AmAp5. Of these, AmAp1∼AmAp3 were isolated from hemolymph of Western honeybees that had been infected with bacteria [21] (Figure 1C). AcAp1 was coded for by 10 different cDNA genes, and AcAp2, AcAp3 and AcAp4 were coded for by one cDNA gene. AcAp1 is identical to AcAp5, and AcAp4 is identical to AmAp3. Compared with other honeybee apidaecin peptides, one amino substitution (Ser-Ile) exists at position 8 in AcAp2, one substitution (Asn-Ile) at position 8 in AcAp3, and one special substitution (Ser-Pro) at position 9 in AmAp4 [28] (Figure 1C). AmAp4 is another apidaecin peptide in the Western honeybee, but it has never been detected in nature [29]. AmAp5 (GenBank: ABY84358), an apidaecin peptide, is unique in the Western honeybee, in that it consists of 17 amino acids and has a significantly different amino acid composition compared with other apidaecin peptides. It was successfully produced in the eukaryocyte expression vector *Lactococcus lactis* [30].

**Figure 4:**
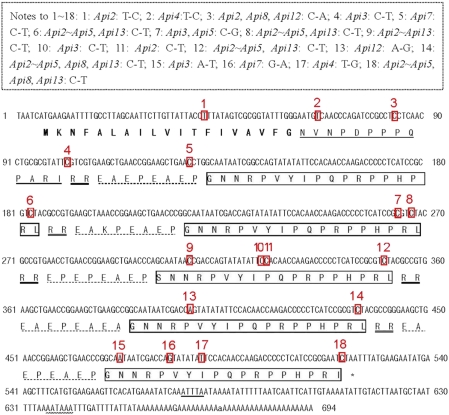
Nucleotide sequence and predicted amino acid sequence of *Apidaecin1* (GenBank Accession EU727255). The putative signal peptide is bolded, the proregion of the peptide indicated by a single line, the dipeptide indicated by double line, the spacer sequences indicated by dotted line, the mature indicated by an open box, the translational signal (TAA) indicated by the star symbol, restriction and poly(A) adenylation signals(AATAAA) indicated by a wavy line, and the ATTTA sequence is indicated by a double wavy line. The variant loci are boxed in red or indicated by a short red arrow, and numbered in red. The nucleotide substitution sites in the precursor sequences between *Apidaecin1* and other *Apidaecin* genes are boxed with a dotted line. “*Apidaecin1∼Apidaecin13*” is abbreviated to “*Abi1∼Api13*”.

The Asiatic honeybee apidaecin precursor gene is composed of two parts: a pre-pro-region and a variable number of repeated, 84 nt long, almost identical units (Figure 4). The pre-pro-region consists of a pre-region (coding for19 amino acids) and a pro-region (coding for 13 amino acids). Each unit consists of coding regions for a spacer sequence and a basic dipeptide (RR) sequence, followed by coding sequence for the mature peptide. All the repeated units in the precursor gene of the Asiatic honeybee contain the same basic dipeptide (RR), except *Apidaecin3*, the nucleotide sequence of the dipeptide (CGT CGT) is mutated to TGT TGT, which codes for another dipeptide CR. In all, 117 NS and 162 SS exist in all the precursor genes, 108 NS and 141 SS in the pro-region and the repeated unit region, and 12 NS and 6 SS in the mature region. Most of the substitutions are in the pro-region. The *apidaecin* mRNA transcript continues for about an additional 140 bases after the stop codon (excluding the PolyA) (Figure 4).

### Analysis of *hymenoptaecin* cDNA genes

**Figure 5:**
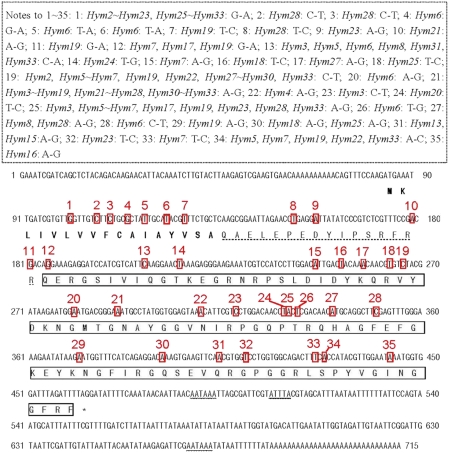
Nucleotide sequence and predicted amino acid sequence of *Hymenoptaecin1* (GenBank Accession EU727297). The putative signal peptide is bolded in was black, the proregion of the peptide is shadowed, and the mature peptide is indicated by an open box. The translational signal (TAG) is indicated by the star symbol. Restriction and poly (A) adenylation signals (AATAAA) are indicated by a wavy line. The ATTTA sequence is indicated by a double wavy line. The variant loci are boxed and numbered in red. The nucleotide substitution sites in the precursor sequences between *Hymenoptaecin1* and other *Hymenoptaecin* genes are boxed with a dotted line. “*Hymenoptaecin1∼Hymenoptaecin34*” is abbreviated to “*Hym1∼Hym34*”.

In all, 34 different *hymenoptaecin* cDNA sequences (*Hymenoptaecin1∼Hymenoptaecin18*, GenBank: EU727297∼EU727314; *Hymenoptaecin19∼Hymenoptaecin34*, GenBank: EU835168∼EU835183) coding for 13 different hymenoptaecin peptides (termed as AcHy1∼AcHy13) were amplified and identified. Compared with the Western honeybee hymenoptaecin peptide (termed as AmHy1, coded for by one *hymenoptaecin* cDNA gene, GenBank: NM 001011615), two amino acid substitutions (Asn-Ser at position 16 and Val-Phe at 85) exist in all the Asiatic honeybee hymenoptaecin peptides, one substitution (Lys-Gln at position 9) in AcHy1∼AcHy5, one substitution (Ala-Ser at position 50) in AcHy1, AcHy2 and AcHy5∼AcHy7, Ser at the same position (position 50) mutates to Thr in AcHy3, AcHy4 and AcHy8∼AcHy13, and one substitution (Ser-Pro at position 46) exists in AcHy2, one substitution (Asn-Asp at position 37) in AcHy9, one substitution (Arg-His at position 53) in AcHy3∼AcHy5, one substitution (Ser-Asn at position 43) in AcHy9, one substitution (Arg-His at position 53) in AcHy3∼AcHy5, one substitution (Ser-Asn at position 43) in AcHy10, one substitution (His-Tyr at position 24) in AcHy11, and one substitution (Leu-Gln at position 50) in AcHy12 (Figure 1D). The Asiatic honeybee hymenoptaecin precursor gene is made up of three parts: a pre-region (coding for 17 amino acids), a pro-region (coding for 16 amino acids) and a mature region (coding for 93 amino acids). In all, 733 NS and 608 SS exist in all the precursor genes, 54 NS and 30 SS in the pre-pro-region, 499 NS and 448 SS in the mature region. The pre-pro-region is highly conserved, and most of the substitutions are in the mature region. The *hymenoptaecin* mRNA transcript continues for about an additional 230 bases after the stop codon (Figure 5), but only 75 bases (excluding the PolyA) after the stop codon are in the *Hymenoptaecin28* sequences.

### Phylogenetic analysis

**Figure 6:**
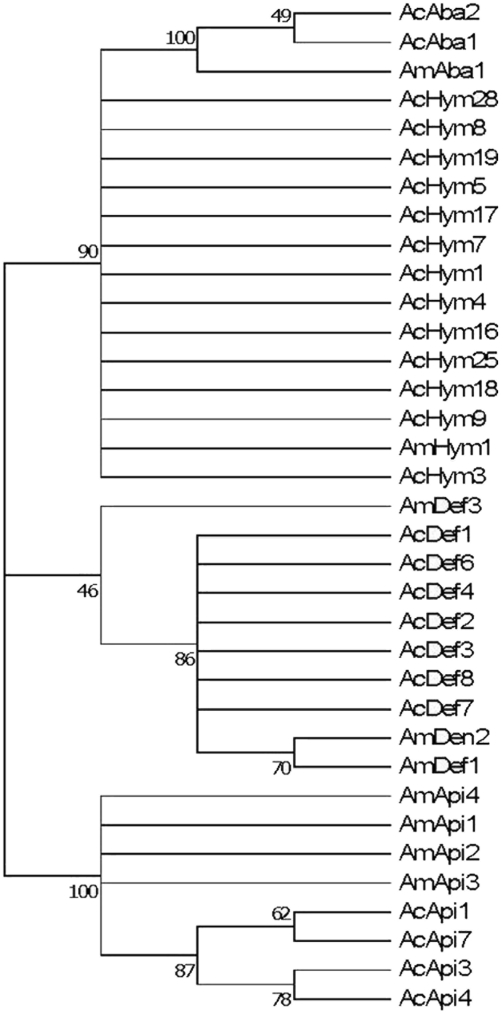
Neighbor-Joining (NJ) tree of four antimicrobial peptide cDNA gene families of *A. cerana* and *A. mellifera*. All the cDNA sequences for *A. cerana* were edited to begin at the initiation codon and end at the stop codon (containing complete cds). All the cDNA sequences for *A. mellifera* are downloaded from the NCBI website and edited to end in the stop codon (containing complete or partial cds). Numbers at nodes are bootstrap values based on 1000 replications. The cDNA gene names, GenBank accession numbers and the encoding peptide names are listed in Table 1.

The different precursor gene sequences from each of the cDNA genes coding for different mature peptides in the Asiatic honeybee and the complete or partial cds sequences in the Western honeybee downloaded from the NCBI website were selected to build the phylogenetic tree. The results show that there are three main branches (*hymenoptaecin*, *defensin* and *apidaecin* families), and the *abacin* cDNA genes have a relatively close relationship with the *hymenoptaecin* cDNA genes (Figure 6). *AmDef3* is one special sequence, which has only 46% possibility to group together with other *defensin* cDNA genes. Because of high homogeneity (similarities ranging from 96.5%∼100%) existing in each of the four antimicrobial peptide gene families, there are only a few branches and nodes within each of the four families (Figure 6). Although a few nucleotide sites vary within each of the four families, even only one nonsynonymous substitution in the relatively variable mature region will induce to generate a new peptide.

## Discussion

As eusocial insects, honeybees have evolved both communal and individual traits that reduce the impact of their numerous parasites and pathogens. Among the individual traits, innate-immune responses have the potential to reduce both individual mortality and the spread of pathogens among colony members [3], [31]. Propolis is a resinous substance variously composed of chewed tree buds mixed with pollen collected by honeybees as well as enzymes secreted by the bees. It has many functions in the defense of the nest, brood and stored food, and plays an important role in protecting the honeybee larvae by preventing diseases and parasites from entering the hive [32]. It's puzzling that the Asiatic honeybee does not produce propolis. How does the Asiatic honeybee protect the larvae? When the workers visit plant flowers to collect pollen and honey, they are inevitably acting as potential disease vectors of different pathogens, such as bacteria, fungus and virus, and even some parasitic mites and insects. Thus the innate immune systems of adult workers are likely to play an important role not only in self defense, but also in helping to insure that the larvae and the queen are shielded from pathogens. In the wild, the Asiatic honeybee may pollinate many more plant species than the Western honeybee, and may therefore be at higher risk of contacting pathogens. It is easy to infer that the Asiatic honeybee may be under stronger selection to defend against pathogens and parasites than the Western honeybee.

Four antimicrobial peptide gene families were isolated from the Western honeybee and identified. Each gene family is responsible for a different duty in the innate immune system of the honeybee. Apidaecin is the most prominent component of honeybee humoral defense. Its unique precursor structure and/or processing makes *apidaecin* easily triggered and potentially overproduced due to a low threshold for transcriptional activation [24]. In contrast, sizable experimental infections are required for induction of the normally silent *defensin*, *abaecin* and *hymenoptaecin* genes. Analysis of the *apidaecin* cDNA sequence of the Western honeybee indicated that up to 12 apidaecin peptides can be generated by processing single precursor proteins [28]. The same results were obtained when analyzing the *apidaecin* cDNA sequence of the Asiatic honeybee. However, only active antimicrobial peptides are detectable in adult honeybee haemolymph [28], [33]. Apidaecin has strong antimicrobial activity to a wide spectrum of common plant-associated gram-negative bacteria [21], [24], and it is the first line of defense against invading pathogens. Abaecin is another major antimicrobial peptide in the haemolymph of the honeybee. It is relatively weaker but as active as apidaecin against both gram-negative and gram-positive bacteria. It also has antimicrobial activity to some bacteria *in vitro* that are resistant to apidaecin [22]. There is no pro-region in the abaecin precursor gene, which means that the active peptide have been produced once it is translocated into the endoplasmic reticulum and without other processing [24]. An additional puzzling feature of abaecin is that it looses most of its antibacterial activity in the presence of phosphate-buffered saline or diluted bee haemolymph, media that resemble its physiological environment [22]. The weakly antimicrobial abaecin seems likely to function as a backup for bacterial resistance against apidaecin [22]. In relation to the major immune component (apidaecin), hymenoptaecin is largely complementary to apidaecin because it inhibits growth of certain gram-negative bacteria that are resistant to apidaecin [23]. Under honeybee physiological conditions, it inhibits the viability of gram-negative and gram-positive bacteria, including several human pathogens [23]. By comparison, bee defensin production is rather minimal and considerably delayed, representing the only component of the honeybee humoral defense specifically targeting gram-positive bacteria [24], [26]. Steady state levels of defensin are probably sufficient to clear persistent gram-positive cells after 24 h of infection [24]. Under minor infection of pathogens in the wild, only *apidaecin* and *hymenoptaecin* genes were detected in the bodies of Western honeybee adults (*abaecin* and *defensin* were absent) [24]. The wide range of gram-negative bacteria targeted by apidaecin may account for the majority of honeybee infections in the wild [24]. Apidaecin and hymenoptaecin peptides might be important factors in restricting or killing different pathogens in the innate immune system of the honeybee. The abaecin peptide seems likely to function as a backup for the potential emergence of bacterial resistance against apidaecin [23]. The defensin peptide may play an important role in maintaining the immunological abilities of honeybees in the later stages of infections.

We have amplified and identified four families of antimicrobial peptide transcript genes from Asiatic honeybee adult workers infected by *E. coli*. Blastn results suggest that all of them share high sequence identity with the Western honeybee. The results of phylogenetic analysis suggest that all the cDNA genes in each of the four gene families in the same honeybee species are paralogous genes, and the peptides they encode for are allelic variants. For example, the high sequence similarities of defensin peptides and the identity of the pre-pro-regions of all peptides suggest that the defensin peptides might be variants encoded for by a single polymorphic gene [25]. In all, at least 29 different *defensin* cDNA genes encoding 7 defensin peptides, although one of the defensin variants (AcDe7) has a substitution at a cysteine residue that is definitive and widely conserved in nature, suggesting that this sequence might be from a pseudogene or allele with null activity. 11 different *abaecin* cDNA genes encoding 2 abaecin peptides, 13 different *apidaecin* cDNA genes encoding 4 apidaecin peptides and 34 different *hymenoptaecin* cDNA encoding 13 hymenoptaecin peptides exist in the Asiatic honeybee. In terms of the Western honeybee, in total 5 *defensin* cDNA genes (containing complete cds sequences, GenBank: NM_001011616, NM_001011638, AY333923, AY588474 and U15955) encoding 3 defensin peptides (AmDe1∼AmDe3), 2 *abaecin* cDNA genes (containing complete cds sequences, GenBank: NM_001011617 and U15954) encoding one 1 abaecin peptide (AmAba1) and 1 directly submitted abaecin peptide (AmAb2, GenBank: AAL35348), 7 *apidaecin* cDNA genes (containing complete mature nucleotide sequences, GenBank: EU382095, NM_001011642, NM_001011613, X72577, X72576, X72575 and AF442148) encoding 5 apidaecin peptides, and 2 *hymenoptaecin* cDNA genes (containing complete cds sequences, GenBank: NM_001011615 and U15956) encoding1 hymenoptaecin peptide have hitherto been reported. It is likely that additional antimicrobial peptide and cDNA genes exist in the Western honeybee, which may encode for the same mature peptides as reported here. However, there is no doubt that more antimicrobial peptides and their cDNA genes are found in the Asiatic honeybee than in the Western honeybee, specifically the hymenoptaecin peptides (13 versus 1). These results suggest that the long history of domestication of *A. mellifera* may have led to a lower standing diversity of antimicrobial peptides in its sequences. Additionally and/or alternatively, Asiatic honeybee adults may be under stronger selection pressure from pathogens and parasites, resulting in greater positive and diversifying selection on antimicrobial peptide sequences than in the Western honeybee.

## Materials and Methods

### Insects

Colonies of the Chinese honeybee, *Apis cerana cerana* Fabricius, have been maintained on the campus of Zhejiang University since 2003. Adult honeybee workers were injected with viable *E. coli* (TG1) as described previously [21]. The fat bodies of the wasp adults were harvested 24 h after injection.

### RT-PCR

Samples of the fat body were collected and washed twice with phosphate-buffered saline (PBS: 140 mM NaCl, 27 mM KCl, 8 mM Na_2_HPO4, 1.5 mM KH_2_PO4, pH 7.4). Total RNA was extracted from 40 live adult workers using a RNeasy® Mini Kit (Qiagen Gmbh, D-40724 Hilden). The cDNA libraries were constructed using a Clontech, SMART™ PCR cDNA Synthesis Kit. After double stranded cDNA was synthesized, it was diluted 100 fold as the template. All the primers were designed from the four types of antimicrobial peptides gene families of *A. mellifera*. Primers used were as follows: P1 (for *abaecin*): GAT CTG CAC ACT CGA GGT CTG (designed from a short stretch of sequence just upstream of the start codon of the *A. mellifera abaecin* gene), PCR conditions were 94°C, 2 min, 40× (94°C, 20 s; 54°C, 30 s; 72°C, 30 S), 72°C, 10 min; P2 (for *apidaecin*): TAA TCA TGA AGA ATT TTG CCT (designed from a short stretch of sequence just upstream of the start codon of the *A. mellifera apidaecin* gene), PCR conditions were 94°C, 2 min, 40× (94°C, 20 s; 54°C, 30 s; 72°C, 40 S), 72°C, 10 min; P3 (for *hymenoptaecin*): GAA ATC GAT CAG CTC TAC AG (designed from a short stretch of sequence just upstream of the start codon of the *A. mellifera hymenoptaecin* gene), PCR conditions were 94°C, 2 min, 40× (94°C, 20 s; 50°C, 30 s; 72°C, 40 S), 72°C, 10 min; P4, P5 and P6 for amplifying complete *defensin* CDS sequences, P4: TGT CGG CCT TCT CTT CAT GG (designed from a short stretch of sequence after the start codon of the *A. mellifera defensin* gene), PCR conditions were 94°C, 2 min, 40× (94°C, 20 s; 58°C, 30 s; 72°C, 30 S), 72°C, 10 min; P5 (for 5′RACE): GAA ACG TTT GTC CCA GAG ATC (designed from a short stretch of sequence just downstream of the sequence amplified by P4), PCR conditions were 94°C, 2 min, 40× (94°C, 20 s; 56°C, 30 s; 72°C, 30 S), 72°C, 10 min; P6 (for 3′RACE): GCC AAT ATG AAG ATC TAT TT (designed from a short stretch near the start codon of the sequence amplified by P5), PCR conditions were 94°, 2 min, 40× (94°C, 20 s; 50°C, 30 s; 72°C, 30 S), 72°C, 10 min. The 5′PCR primer in the kit was used as the forward primer and P5 as the reverse primer for 5′RACE of *defensin*. The CDS primer (ATT CTA GAG GCC GAG GCG GCC GAC ATG) in the kit was used as the reverse primer in all other PCR reactions. The PCR products were gel purified and cloned with TaKaRa pMD19-T Vector (TaKaRa Biotechnology, Dalian, China). Positive clones were first screened in blue/white colony screening, then positive clones were tested again with M13 vector primers in order to avoid, as far as possible, false positive clones. We sent 5∼10 tested positive clones for each antimicrobial peptide gene family each time to Invitrogen Biotechnology Company (Invitrogen, Shanghai, China) for sequencing until no new cDNA sequence (coding different mature peptide) was found. In all, 156 clones that tested positive were sent to sequence, 56 clones for *defensin*, 23 clones for *abaecin*, 25 clones for *apidaecin* and 52 clones for *hymenoptaecin*.

### Sequence analyses

Signal peptide cleavage sites were predicted using the SignalP 3.0 server program [34]. The nonsynonymous substitutions (NS) and synonymous mutations (SS) were analyzed using the software Dambe [35]. Multiple sequence alignment was performed using Clustal_X [36] and edited with software GeneDoc [37]. The phylogenetic tree was constructed by Neighbor-Joining (NJ) analysis using Mega4 [38] based on the sequence data listed in Table 1.

**Table 1:** Four antimicrobial peptide cDNA gene families of *A. cerana* and *A. mellifera* selected to construct the phylogenetic tree.

Honeybee species	cDNA genes	GenBank accession numbers	Encoded peptide
***A. mellifera***	*AmDef1*	U15955	AmDe1
	*AmDef3*	AY588474	AmDe3
	*AmApi1*	NM_001011642	AmAp1
	*AmApi3*	X72577	AmAp1
	*AmHym1*	U15956	AmHym1
	*AmDen2*	AY333923	AmDe2
	*AmAba1*	NM_001011617	AmAb1
	*AmApi2*	NM_001011613	AmAp1
	*AmApi4*	AF442148	AmAp1
***A. cerana***	*AcDef1*	EU727268	AcDe1
	*AcDef6*	EU727273	AcDe3
	*AcDef8*	EU727275	AcDe5
	*AcDef7*	EU727274	AcDe7
	*AcAba1*	EU714043	AcAb2
	*AcApi3*	EU727257	AcAp2
	*AcApi7*	EU727261	AcAp4
	*AcHym3*	EU727299	AcHy2
	*AcHym5*	EU727301	AcHy4
	*AcHym8*	EU727304	AcHy6
	*AcHym16*	EU727312	AcHy8
	*AcHym18*	EU727314	AcHy10
	*AcHym28*	EU835177	AcHy12
	*AcDef4*	EU727271	AcDe2
	*AcDef3*	EU727270	AcDe4
	*AcDef2*	EU727269	AcDe6
	*AcAba2*	EU714044	AcAb1
	*AcApi1*	EU727255	AcAp1
	*AcApi4*	EU727258	AcAp3
	*AcHym1*	EU727297	AcHy1
	*AcHym4*	EU727300	AcHy3
	*AcHym7*	EU727303	AcHy5
	*AcHym9*	EU727305	AcHy7
	*AcHym17*	EU727313	AcHy9
	*AcHym25*	EU835174	AcHy11
	*AcHym19*	EU835168	AcHy13

## References

[1] Poinar, Danforth BN (2006). A fossil bee from early cretaceous Burmese amber.. Science.

[2] The Honeybee Genome Sequencing Consortium (2006). Insights into social insects from the genome of the honeybee *Apis mellifera*.. Nature.

[3] Breed MD, Guzmán-Novoa E, Hunt GJ (2004). Defensive behavior of honey bees: organization, genetics, and comparisons with other bees.. Annual Review of Entomology.

[4] Chen YP, Evans JD (2007). Historical presence of Israeli acute paralysis virus in the United States.. American Bee Journal.

[5] Chen YP, Evans JD, Smith IB, Pettis JS (2008). *Nosema ceranae* is a long-present and wide-spread microsporidian infection of the European honey bee (*Apis mellifera*) in the United States.. Journal of Invertebrate Pathology.

[6] Higes M, Martín R, Meana A (2006). *Nosema ceranae*, a new microsporidian parasite in honeybees in Europe.. Journal of Invertebrate Pathology.

[7] Klee J, Besana AM, Genersch E, Gisder S, Nanetti A (2006). Widespread dispersal of the microsporidian *Nosema ceranae*, an emergent pathogen of the western honey bee, *Apis mellifera*.. Journal of Invertebrate Pathology.

[8] Santillán-Galicia MT, Carzaniga R, Ball BV, Alderson PG (2008). Immunolocalization of deformed wing virus particles within the mite *Varroa destructor*.. Journal of General Virology.

[9] Yang GH (2005). Harm of introducing the western honeybee *Apis mellifera* L. to the Chinese honeybee *Apis cerana* F. and its ecological impact.. Acta Entomologica Sinica.

[10] From Wikipedia, the free encyclopedia.. http://en.wikipedia.org/wiki/Apis_cerana.

[11] Kasprzak S, Topolska G (2007). *Nosema ceranae* (Eukaryota: Fungi: Microsporea) —a new parasite of western honey bee *Apis mellifera* L.. Wiad Parazytol.

[12] Williams GR, Shafer AB, Rogers RE, Shutler D, Stewart DT (2008). First detection of *Nosema ceranae*, a microsporidian parasite of European honey bees (*Apis mellifera*), in Canada and central USA.. Journal of Invertebrate Pathology.

[13] Souza RM, de Souza MC, Patitucci ML, Silva JF (2007). Evaluation of antioxidant and antimicrobial activities and characterization of bioactive components of two Brazilian propolis samples using a pKa-guided fractionation.. Zeitschrift fur Naturforschung C-A Journal of Biosciences.

[14] Fontana R, Mendes MA, de Souza BM, Konno K, César LMM (2004). Jelleines: a family of antimicrobial peptides from the royal jelly of honey bees (*Apis mellifera*).. Peptides.

[15] Girardin SE, Sansonetti PJ, Philpott DJ (2002). Intracelluar vs extracellular recognition of pathogens—common concepts in mammals and flies.. Trends in Microbiology.

[16] Loker ES, Adema CM, Zhang SM, Kepler TB (2004). Invertebrate immune systems—not homogenous, not simple, not well understood.. Immunological Reviews.

[17] Müller U, Vogel P, Alber G, Schaub GA (2008). The innate immune system of mammals and insects.. Contributions to Microbiology.

[18] Boman HG (1995). Peptide antibiotics and their role in innate immunity.. Annual Review of Immunology.

[19] Bulet P, Hetru C, Dimarcq J-L, Hoffmann D (1999). Antimicrobial peptides in insects: structure and function.. Developmental and Comparative Immunology.

[20] Hoffmann JA, Kafatos FC, Janawey CA, Ezekovitz RAB (1999). Phylogenetic perspectives in innate immunity.. Science.

[21] Casteels P, Ampe C, Jacobs F, Vaek M, Tempst P (1989). Apidaecins: antimicrobial peptides from honeybees.. EMBO Journal.

[22] Casteels P, Ampe C, Riviere L, Damme JV, Elicone C (1990). Isolation and characterization of abaecin, a major antimicrobial peptide in the honeybee (*Apis mellifera*).. European Journal of Biochemistry.

[23] Casteels P, Ampe C, Jacobs F, Tempst P (1993). Functional and chemical characterization of hymenoptaecin, an antimicrobial peptide that is infection-inducible in the honeybee (*Apis mellifera*).. Journal of Biological Chemistry.

[24] Casteels-Josson K, Zhang W, Capaci T, Casteels P, Tempst P (1994). Acute transcriptional response of the honeybee peptide-antibiotics gene repertoire and required post-translational conversion of the precursor structures.. Journal of Biological Chemistry.

[25] Klaudiny J, Albert Š, Bachanová K, Kopernický J, Šimúth J (2005). Two structurally different defensin genes, one of them encoding a novel defensin isoform, are expressed in honeybee *Apis mellifera*.. Insect Biochemistry and Molecular Biology.

[26] Fujiwara S, Imai J, Fujiwara M, Yaeshima T, Kawashima T (1990). A potent antibacterial protein in royal jelly.. Journal of Biology Chemistry.

[27] Eipper BA, Mains RE, Glembotski CC (1983). Identification in pituitary tissue of a peptide α-amidation activity that acts on glycine-extended peptides and requires molecular oxygen, copper, and ascorbic acid.. Proceedings of the National Academy of Sciences of the United States of America.

[28] Casteels-Josson K, Capaci T, Casteels P, Tempst P (1993). Apidaecin multipeptide precursor structure: a putative mechanism for amplification of the insect antibacterial response.. EMBO Journal.

[29] Casteels P, Romagnolo J, Castle M, Casteel-Josson K, Erdjument-Bromage H (1994). Biodiversity of apidaecin-type peptide antibiotics: Prospects of manipulating the antibacterial spectrum and combating acquired resistance.. Journal of Biological Chemistry.

[30] Zhou X, Wang Y, Pan Y, Li W (2008). Nisin-controlled extracellular production of apidaecin in *Lactococcus lactis*.. Applied Microbiology and Biotechnology.

[31] Evans JD, Pettis JS (2005). Colony-level impacts of immune responsiveness in honey bees, *Apis mellifera*.. Evolution.

[32] From Wikipedia, the free encyclopedia.. http://en.wikipedia.org/wiki/Propolis.

[33] Li WF, Ma GX, Zhou XX (2006). Apidaecin-type peptides: Biodiversity, structure-function relationships and mode of action.. Peptides.

[34] SignalP 3.0 Server.. http://www.cbs.dtu.dk/services/SignalP/.

[35] Xia X, Xie Z (2001). DAMBE: software package for data analysis in molecular biology and evolution.. Journal of Heredity.

[36] Thompson JD, Gibson TJ, Plewniak F, Jeanmougin F, Higgins DG (1997). The CLUSTAL_X windows interface: flexible strategies for multiple sequence alignment aided by quality analysis tools.. Nucleic Acids Research.

[37] Nicholas KB, Nicholas HBJ, Deerfield DW (1997). GeneDoc: Analysis and visualization of genetic variation.. EMBNEW News.

[38] Tamura K, Dudley J, Nei M, Kumar S (2007). MEGA4: Molecular evolutionary genetics analysis (MEGA) software version 4.0.. Molecular Biology and Evolution.

